# Predictors of Preeclampsia Based on a 10-Year Case-Control Study

**Published:** 2019-03

**Authors:** Arezoo Shayan, Hadis Sourinejad, Mansoureh Refaei, Seyedeh Zahra Masoumi, Leili Tapak, Farzaneh Soltani

**Affiliations:** 1 Mother and Child Care Research Center, Hamadan University of Medical Sciences, Hamadan, Iran; 2 School of Nursing and Midwifery, Hamadan University of Medical Sciences, Hamadan, Iran; 3 Department of Biostatistics, School of Public Health, Hamadan University of Medical Sciences, Hamadan, Iran

**Keywords:** Preeclampsia, Prediction, Seasonal Changes, Blood Group

## Abstract

**Objective:** To investigate the predictors of preeclampsia based on a 10-years case-control study.

**Materials and methods:** The present retrospective, case-control study was carried out in a referral Hospital in Hamadan, Iran, during 2005-2015. Using a hospital information system (HIS), all the available information on hospitalized preeclamptic patients during 10-years period was collected and all preeclamptic women were qualified for the study as the case group (729 subjects) and the same number of non-preeclamptic women were assigned to the control group. The required data were collected using the researcher-made questionnaire and analyzed using descriptive statistics, chi-square test, independent t-test and logistic regression.

**Results:** The results show that high age (OR = 1.04), low education (diploma compared to illiterate OR = 0.51 and middle school education compared to illiterate OR = 0.55), blood group O (AB: OR = 0.32; B: OR = 0.36; A: OR = 0.26) and fertilization during autumn (spring: OR = 0.41; summer: OR = 0.26; autumn: OR = 1.13) could raise the risk of preeclampsia.

**Conclusion:** High age, low education, blood group O and fertilization in cold seasons could be the risk factors of preeclampsia. Recognizing the risk factors of preeclampsia could help the determination of high risk cases and designing of effective interventions.

## Introduction

Hypertensive disorders are one of the deadly triad along with hemorrhage and infection. Preeclampsia is a pregnancy specific syndrome in which the perfusion of organs is deteriorated due to vasospasm and endothelial activation, and consequently, organs dysfunction occurs (1). Although the prevalence of preeclampsia is 3 to 10% globally, it has been reported as high as 20% in developing countries (2). The World Health Organization estimates that preeclampsia contributes directly to 10% of maternal mortality in Asia (3). Kharaghani et al. estimated the prevalence of preeclampsia in Iran to be 5% (4). Moreover, in another study in Tehran, the prevalence of preeclampsia was reported as 6.4% (5). Despite much advancement in the clinical management of preeclampsia, its etiology, as one of the most critical subjects in the field, is still unclear; thus, preeclampsia is regarded as a typical “disease of theories”. However, the pre-diagnosis of patients is very important due to the serious complications of this disease such as placental abruption, acute renal failure, cerebrovascular accidents, disseminated intravascular coagulation (DIC), and maternal death. Despite numerous studies on preeclampsia screening tests, there is no reliable, authentic and affordable screening test for preeclampsia. However, preeclampsia can be detected at early stages only with planned prenatal cares. The evidence suggests that the dysfunction starts at early stages of pregnancy and hidden pathophysiologic changes start at the time of fertilization (6). Thus, as strong hypotheses certain preconceptional agents are correlated with the high risk of disease. In the past, the researchers have found that change in temperature, seasonal change and rainfall were correlated with the disease and several studies have shown that there is a relationship between the incidence of preeclampsia and the season at delivery. For example, a 30-year study in Norway showed that there is higher incidences of preeclampsia among women who gave birth in the winter than in the summer (7). In addition, Chesley reported a higher rate of preeclampsia in June marriages in New York (8). However, it is yet unclear whether the time of fertilization is correlated with preeclampsia or the time of delivery. In response to the above question, Phillip et al. studied 7904 pregnant women with preeclampsia and found that there was a stronger correlation between preeclampsia and fertilization season than birth season, that is, fertilizations in the summer had higher risk of preeclampsia incidence (70%) compared with fertilizations in spring (9). Nevertheless, there is contradiction between the results of studies on how changes in season or climate affect the incidence of preeclampsia (10, 11). Moreover, there are contradictions among studies on the correlation between preeclampsia and maternal blood group (12). The correlation between ABO/Rh blood groups and preeclampsia has been a controversial topic for many years. For example, South and Naldrett found no correlation between the risk of preeclampsia and blood groups O and A (13); however, May reported a high risk of preeclampsia in blood group A (14). In two studies with a two-year interval in Brazil, Hentschke et al. found no differences in distribution between blood groups in two case and control groups (15, 16). On the other hand, the results of recent studies have showed a correlation between blood group AB and higher risk of preeclampsia (17, 18). However, given the fixed blood groups in the human lifetime, it is very important to determine the correlation between preeclampsia and blood groups. Identification the predictors of hypertensive disorders in pregnancy can have a significant role in reducing maternal and fetal mortality and morbidity; therefore, the present study was conducted to investigate the predictors of preeclampsia to make possible the provision of special cares for high risk women through implementing such studies.

## Materials and methods

The present retrospective, case-control study was carried out using a hospital information system (HIS) at Fatemieh Hospital in Hamadan, Iran for a decade. The study inclusion criteria included all primigravida cases hospitalized after 20 weeks of pregnancy, diagnosed with preeclampsia in the hospital during 2005-2015, admitted with a complete medical file and accurate LMP. The study exclusion criteria included background diseases, incomplete medical file and uncertain date of last menstruation. Diagnostic criteria of preeclampsia were a systolic blood pressure ≥ 140 mmHg or diastolic blood pressure ≥ 90 mmHg as measured twice after 20 weeks of pregnancy along with proteinuria of over 300 mg/l in 24-hour urine test (1). At First, all the codes of the pregnant women hospitalized during 2005-2015 were extracted. Then, the files with the preeclampsia code were picked out and the history sheet was examined. The eligible cases were selected based on the Initial and final diagnosis of preeclampsia. Out of 80,000 birth cases during the decade, 726 preeclampsia cases were assigned to the case group and 726 non-preeclamptic pregnant cases were assigned to the control group. The collected data were analyzed using descriptive statistics and logistic regression in SPSS software (version 24, IBM Corporation, Armonk, NY, USA), and P-values less than 0.05 were considered significant.

## Results

Table 1 shows the comparison of demographic and obstetric characteristic of women in the preeclampsia and control groups. The majority of the patients were housekeeper and did not have a job (96.1% of preeclampsia and 97.5% of controls), had diploma (30.9% of preeclampsia and 52.2% of controls) and aged between 31 to 40 (67.5% of preeclampsia and 58.1% of controls). In preeclampsia and control groups, 58.5% and 86% of participants were primigravida cases, respectively, and this difference was statistically significant (p < 0.001). 

**Table 1:** Comparison of demographic and obstetric characteristic of women in the preeclampsia and control groups

		Preeclampsia		Control		**ꭕ** **2**test	P-value
		**Number**	**Percent**	**Number**	**Percent**		
Parity	0	430	59.2	627	86.4	138.02	*< 0.001
	1	170	23.4	55	7.6		
	2	91	12.5	28	3.9		
	3	20	2.8	8	1.1		
	4 <	15	2.1	8	1.1		
	Total	726	100	726	100		
Gravidity	1	425	58.5	624	86.0	144.71	*< 0.001
	2	142	19.6	48	6.6		
	3	90	12.4	26	3.6		
	4	39	5.4	15	2.1		
	5	17	2.3	7	1,0		
	+6	13	1.8	6	1.6		
	Total	726	100	726	100		
Age (year)	20 >	33	4.5	48	6.6	50.57	*< 0.001
	21-30	163	22.5	137	18.9		
	31-40	490	67.5	422	58.1		
	41-50	39	5.4	109	15		
	50 <	1	0.1	10	1.4		
	Total	726	100	726	100		

* p < 0.05

Table 2 illustrates the frequency of different blood groups for preeclamptic cases and controls. According to the table, there was a significant difference in terms of blood group and RH distribution between two groups (p < 0.001). Most preeclamptic cases (60.2%) were of the blood group O^+^; however, most cases in the control group (56.1%) were of the blood group A^+^. Furthermore, blood group distribution alone showed a significant difference between the groups regardless of RH (p < 0.001). However, the majority of women in the preeclampsia group (63.4%) were of the blood group O, but women of the blood group A were the majority (58.7%) in the control group (Table 2).

**Table 2:** Comparison of blood groups and RH in the preeclampsia and control groups

		**Preeclampsia**			**Control**		ꭕ2**test**	**P-value**
		**Number**	**Percent**	**Number**		**Percent**		
Blood Group/Rh	A+	117	16.1		407	56.1	340.41	*< 0.001
	A-	9	1.2		19	2.6		
	B+	91	12.5		87	12.0		
	B-	10	1.4		8	1.1		
	AB+	36	5.0		48	6.6		
	AB-	3	0.4		0	0.0		
	O+	437	60.2		127	17.5		
	O-	23	3.2		30	4.1		
	Total	726	100		726	100		
Blood Group	A	126	17.4		426	58.7	90.07	*< 0.001
	B	101	13.9		95	13.1		
	AB	39	5.4		48	6.6		
	O	460	63.4		157	21.6		
	Total	726	100		726	100		

* p < 0.05

**Table 3:** Estimation of parameters and chance ratios in terms of Fit logistic regression model

**Variable**	**Estimated**	**Standard error**	**P-value**	**OR (95% CI)**
Invariant	4.027	1.604	*0.012	56.078
Age	0.040	0.010	*0.001	1.04 (1.05,1.02)
Blood Group				
O	-	-	-	1
A	-2.406	0.169	*0.001	0.09 (0.13,0.06)
B	-1.034	0.207	*0.001	0.36 (0.53,0.24)
AB	-1.125	0.278	*0.001	0.32 (0.56,0.19)
Fertilization season				
Spring	-0.897	0.205	*0.001	0.41 (0.61,0.27)
Summer	-1.365	0.204	*0.001	0.26 (0.38,0.17)
autumn	0.124	0.224	*0.001	1.13 (1.76,0.73)
winter	-	-	-	1
Level of Education				
Illiterate	-	-	-	1
Elementary	-0.099	0.294	0.738	0.91 (1.96,0.62)
under Diploma	-0.602	0.245	*0.014	0.55 (0.88,0.34)
Diploma	-0.608	0.237	*0.004	0.51 (2.63,0.68)
Academic	0.288	0.347	0.406	1.33 (2.63,0.68)
Nulliparous	0.381	0.244	0.118	1.46 (0.98,1.94)
Primigravida	0.118	0.199	0.554	1.12 (0.729,1.51)

* p < 0.05

Significant difference in the distribution of fertilization month between the two groups (p < 0.001). Most mothers in the preeclampsia group (15%) and control group (18.9%), respectively, gave birth in March and September. In addition, there was a significant difference in fertilization season distribution between the groups (p < 0.001). Most mothers in the preeclampsia group (54%) gave birth in the second half of the year (autumn and winter) and most mothers in the control group (72.6%) gave birth in first half of the year (summer).

Table 3 shows the results of the fit logistic regression model in the examination of the modified effects of variables on the preeclampsia chance logarithm. The table illustrates the estimation of parameters and odds ratios obtained from logistic regression as well as their P-values. The positive coefficient illustrates the direct effect on preeclampsia chance logarithm. In the examination of the effects of qualitative variables, one level is considered as the basic level and the estimation of effect is regarded as a comparison of group(s) in terms of basic level. The value of 1 illustrates the ineffectiveness of the variable. Age had a significant impact on the risk of preeclampsia (p < 0.001) and increase in age resulted in an increase in odds of preeclampsia, that is, with every one year increase in age there is a 4% increase in odds of preeclampsia. Moreover, having the blood group O increased the chance of preeclampsia, so that people of the blood group O had a greater chance (1/0.09) than the people with blood group A (11.11 times higher). The fertilization season had a significant impact on chance of preeclampsia incidence (p < 0.001). However, fertilization in autumn rather than in spring increases the odds of preeclampsia as the odds of fertilization in autumn was 2.44 (= 1/0.41) times greater than fertilization in spring. Furthermore, education had a significant impact on the chance of preeclampsia incidence (p < 0.001) and people with a diploma had less chance of preeclampsia incidence than those who were illiterate. Therefore, the chance of preeclampsia in illiterate women was 1.96 (= 1/0.51) times greater than the women who had a diploma.

The results of the goodness of fit test (Table 4) approve the adequacy of the twin logistic regression model based on deviance criterion (p = 0.412). Furthermore, sensitivity and specificity, positive and negative predictive value, general accuracy and area under the ROC Curve were calculated for the model (AUC=). The fitted model had adequate sensitivity (0.810) and specificity (0.807).

**Table 4:** The result of the Hosmer-Lemeshow Test for the goodness of fit logistic regression model

**Chi-square statistics**	**Df**	**P-value**
8.223	8	0.412

## Discussion

The results of the present study showed that high age, low education, blood group O and fertilization in autumn increase the risk of preeclampsia. Most preeclamptic cases were in the range of 39-40 years of age. Age of higher than 35 years is a risk factor for preeclampsia (19). Magnus and Eskild, in their 30-year study in Norway, found that pregnant women in the range of 35-39 years of age are at a 1.8 times greater risk of preeclampsia than their younger counterparts and this risk raised to 2.4 times higher for women of over 40 years of age (7). In addition, low education of pregnant women, as an indicator of low socioeconomic class, in other studies has been associated with an increased risk of preeclampsia. Other researchers have found association between low educational attainment, especially in low-income countries, with an increased risk of preeclampsia (20, 21). In the present study the highest incidence of preeclampsia was observed in pregnancies fertilized in March and the lowest in July, and incidence of preeclampsia was reduced by fertilization in winter, autumn, spring, and summer respectively, In other words, the incidence of preeclampsia is increased in cold climates and decreased in warm weather. Some studies reported the highest incidence of preeclampsia in pregnancies fertilized during cold seasons and the lowest during summer (22-24). Magnus et al. in a retrospective 30-years study during 1967-1998 on 1,869,388 births in Norway, found that mothers who gave birth in August were at the least risk of preeclampsia incidence, but those who gave birth in December were at the highest risk of preeclampsia incidence (7). Although, the criteria for the mentioned study were the month of delivery, it can be easily found that the fertilization of mothers who had given birth in December had been around March of the previous year, which is consistent with the results of our study that preeclampsia is associated with fertilization in cold seasons. In fact, the studies performed in the recent decade have provided a better understanding of the potential mechanisms of preeclampsia pathogenesis. It was assumed for a long time that cold weather causes vasospasm followed by ischemia which forms part of the pathogenesis of preeclampsia (25). The preconceptional factors, especially the plasma volume and vascular tonicity, have been raised in this regard. It is assumed that the onset of the preeclampsia is secondary reduction of placental uterine perfusion to abnormal cytotrophoblastic invasion of spiral arterioles. Thus, placental hypoxia/ischemia causes extensive dysfunction/activation of maternal vascular endothelium followed by formation of endothelia, thromboxane and superoxide, vascular hypersensitivity to angiotensin II, Reduction in prostacyclin production and the result of this abnormal vascular changes is increased peripheral resistance and hypertension. Researchers who have found the higher risk of preeclampsia in pregnancies with fertilization in the cold seasons, have discussed about numerous possible mechanisms such as changes in climate, temperature and humidity, infection and seasonal variation in food intake (25-27). Accordingly, vasoconstriction in cold seasons (28), fluctuations in the prevalence of seasonal infections which cause inflammatory responses in pregnancy, or changes in maternal diet during different seasons such as antioxidants and calcium, and low intake of vitamin D in cold seasons (29), have been discussed. On the other hand, studies that reported a high risk of preeclampsia in pregnancies with fertilization during the summer, have suggested changes in blood plasma volume influenced by climate changes (9) or heat-affected vascular damage in the primary stages of pregnancy and placenta implantation process (30). ‎Zahirisoroori ‎et al. in Guilan (the North of Iran) did not find a significant relationship between preeclampsia / eclampsia, and seasons (29). They stated that it was not possible to confirm the study results due to the lack of sufficient distinction between seasons (for example, relative similarity between spring and summer or autumn and winter) and lack of temperature and humidity differentiation throughout the year in this area. (29) It should be noted that though Hamadan is regarded as one of the cold region in Iran, it has four distinct and separate seasons which puts the present study in a unique situation in this regard. In our study, the risk of developing preeclampsia was significantly higher in blood group O^+ ^than other blood groups. Different studies reported contradictory results on this matter. The findings of Elmugabil et al. support the present study results (31). Hentschke et al. found no relationship between blood groups and preeclampsia (16). Since a hemostatic abnormality in the placental circulation of preeclamptic patients has been shown for many years, it can be assumed that the relationship of blood groups with preeclampsia can reflect the multifactorial characteristics of clot formation in the placenta (32). It seems that, due to the multifactorial nature of preeclampsia, in addition to blood groups, other thrombophilic factors may contribute to develop disease (33). Conducting longitudinal studies in larger populations and in different communities to investigate the risk factors of preeclampsia could help the determination of high risk pregnant women and designing of effective interventions.

## Conclusion

According to the results of the present study, higher age, lower education, blood group O^+^ and fertilization in cold seasons could be the risk factors of preeclampsia.
